# Soldiers Initiate Foraging Activities in the Subterranean Termite, *Heterotermes tenuis*

**DOI:** 10.1673/031.008.0201

**Published:** 2008-01-09

**Authors:** Fabiana Elaine Casarin, Ana Maria Costa-Leonardo, Alberto Arab

**Affiliations:** Department of Biology, Institute of Biosciences, Unesp, Rio Claro, SP, Brazil

**Keywords:** termites, *Heterotermes tenuis*, soldier, foraging behavior, polyethism

## Abstract

Caste polyethism has been recorded in some termite species, however the foraging behavior of subterranean termites remains poorly known. *Heterotermes tenuis* Hagen (Isoptera: Rhinotermitidae) is a subterranean termite that is native to Brazil and is an agricultural and urban pest. The aim of this study was to investigate which caste acts as scouts when searching for food sources and determinate the percentages of each caste present in the foraging territories of field colonies of *H. tenuis. *Our results showed no significant differences among the caste proportions present in the foraging territories of the three colonies studied in the field. Laboratory experiments showed that minor soldiers were the most frequent initiators of foraging activities. This result suggests that the exploratory phase of the foraging behavior may be regulated by the number of soldiers present in the foraging territories of each colony.

## Introduction

Termite foraging is a social process in which groups of individuals search in organized patterns for new food sources (Traniello and Leuthold 2000). This process is poorly understood in several species, especially in subterranean termites that possess cryptic habits. Predictable and different phases occur during termite foraging . The first phase corresponds to exploration of the foraging area, and the second phase is characterized by the recruitment of other individuals. The exploration phase involves few termites (workers or soldiers) that act as scouts (Reinhard et al. 1997). The recruitment of other individuals starts when the first scout returns to the nest after discovering a new food source and the second phase involves larger numbers of termites (Schedel and Kaib 1987).

The soldier caste of termites is unique among social insects by its morphology, development and behavior (Noirot 1990). Soldiers may derive from all worker instars, pseudergates or apterous immature forms. This polymorphic origin of soldiers is responsible for the dimorphic or even trimorphic forms of this caste in some termite species, that belong to the families Kalotermitidae, Rhinotermitidae and Termitidae (Roisin 2000).

The behavior of termite soldiers is extremely simple when compared to that of other members of the colony, since they are primarily specialized in the function of nest defense (Noirot 1990). Nevertheless, in some species, soldiers also play a role as scouts by exploring new food sources (Traniello 1981; Kaib 1985), although this characteristic appears to be a recent acquisition (Noirot 1990).

*Heterotermes tenuis* Hagen 1858 (Isoptera: Rhinotermitidae) is native to Brazil, where it is considered an agricultural pest of economic importance because it causes considerable damage to sugarcane crops (Pizano and Fontes 1986). *H. tenuis* also infests buildings where it feeds on wood or other cellulosic materials (Gonçalves and Silva 1962). This subterranean termite has a dimorphic soldier caste (Constantino 2000), but the role of each soldier type during foraging activities in this species is unknown. The nest of *H. tenuis* is constituted of a simple network of underground galleries plastered with faecal material, and the foragers generally leave this diffuse nest to search for cellulosic resources above ground (Costa-Leonardo 2002). The aim of this study was to investigate which caste acts as scouts when searching for food sources and to determinate the percentages of each caste present in the foraging territories of field colonies of *H. tenuis*.

## Materials and methods

### Caste percentages in foraging territories

Foraging termites of *H. tenuis* were captured in underground cardboard traps over 12 months from three field colonies (A, B, and C). The colonies were located in Rio Claro (22° 23′ 43″ S, 47° 32′ 39″ W), Sao Paulo, Brazil. Three monitoring stations were randomly placed in the foraging territories of each colony, and the trap that captured the most termites was chosen for evaluating the percentages of each caste involved in foraging activities. Three randomly chosen subsamples (2 ml) of the total amount of the captured termites of each colony were isolated and separated by caste. This percentage value for each caste was square root arcsine transformed before the statistical analyses. Two-way ANOVA (P<0.05) was employed to compare the means of each caste of the three colonies, and the Tukey Multiple Comparison Test (P<0.05) was used to detect which were different (Sokal and Rohlf 1995).

### Monitoring foraging behavior in the laboratory

Laboratory experiments were conducted at 24–26°C and 80% RH, using 15-cm Petri dishes as test chambers, in which was placed 10 g of moistened sterilized sand as tunneling substrate to be used as an artificial nest by the termites. After a 24-hour adaptation period, pinewood sawdust was placed on the opposite side of the sand inside the Petri dish as a food source. Three bioassays were performed with different percentages of soldiers and workers. Ten replications were used for each bioassay. Bioassays 1 and 2 were based on the same proportion of workers (95%) and soldiers (2% of major soldiers and 3% minor soldiers) found in the field colonies. The total amount of individuals in the test chambers was varied, i.e., 100 individuals in bioassay 1 and 200 individuals in bioassay 2. In bioassay 3, the percentage of soldiers was increased up to 20% (10% major soldiers and 10% minor soldiers) and 200 individuals were confined in the test chambers. After placing the food source in the test chambers, the experiments were tape-recorded and the first individual to reach the food was registered in each bioassay. The results were subjected to an analysis of frequencies using G-test (P<0.01) (Sokal and Rohlf 1995).

**Table 1.**
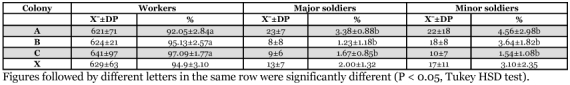
Means and percentages ± SD of the castes found at the foraging territories of colonies of *H. tenuis* in the field.

**Table 2.**
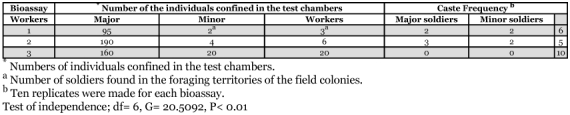
Frequencies of the individuals that initiated the exploratory phase of the foraging behavior in *H. tenuis*.

## Results

**Figure 1.**
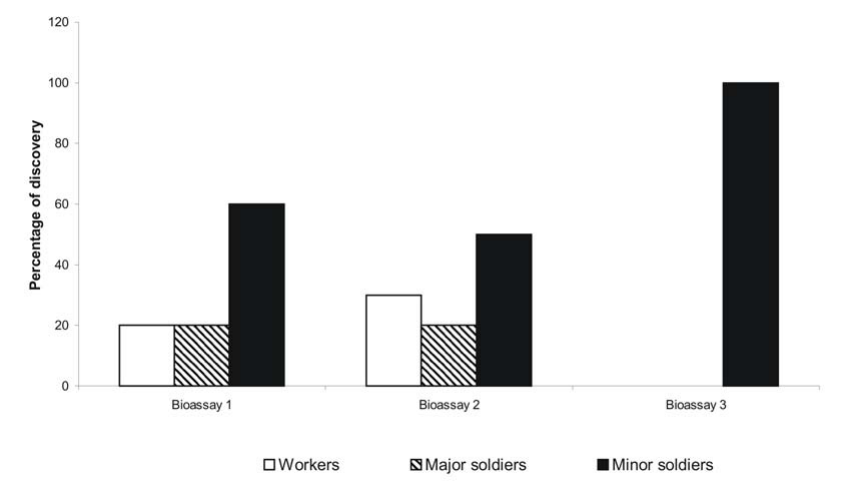
Mean percentage (± SD) of each caste initiating the exploratory phase of the foraging behavior in *H. tenuis*.

The percentages of each caste found in the foraging territories of the field colonies of *H. tenuis* were not significantly different for the three colonies (ANOVA, F= 0.168, df= 2, P= 0.846) (Table 1). Workers were the most abundant caste in all colonies (ANOVA, F= 2489.81, df= 2, P= 0.0001) and no differences were found between the percentages of major and minor soldiers (Tukey HSD, P> 0.05) (Table 1). Termites confined in the test chambers for 24 hours remained inside the sand until the food source was added, when the soldiers started acting as scouts in most of the replicates. Minor soldiers were the individuals that initiated foraging behavior most frequently in all bioassays (Table 2). The results from bioassays 1, 2 and 3 show that the frequency of the castes in the foraging activity was dependent on the number of soldiers inside the test chambers (Test of independence; df= 6, G= 20.5092, P< 0.01) (Figure 1, Table 2). On the other hand, the frequency of each caste in foraging activities was independent from the total number of termites inside the test chambers (Test of Independence, df= 2, G= 0.14619, P> 0.01). When the percentage of soldiers was increased up to 20% (Bioassay 3), only minor soldiers initiated the exploratory phase of foraging (Figure 1, Table 2). The massive recruiting of workers and soldiers only took place when a scout returned to the “sand nest” after finding the food source, after which the workers built shelter galleries linking the sand to the food source.

## Discussion

Soldier polymorphism seems often correlated with division of labor, i.e. polyethism, but this problem remains poorly explored in Isoptera and it follows different patterns inside of the same genus (Noirot and Darlington, 2000).

Caste polyethism in subterranean termites has been poorly studied because, in most cases, they forage inside shelter tubes, underground galleries, and even inside the wood (Miura and Matsumoto 1998). Therefore, most food-seeking activities take place out of the sight of the observers (Traniello and Leuthold 2000). Our results illustrated that minor soldiers of *H. tenuis* act as scouts during the exploratory phase of foraging in the laboratory simulations, leading the workers and others soldiers to the food source. Similar behavior was described for the rhinotermitid *Schedorhinotermes lamanianus, *in which the minor soldiers initially recruit some workers and stimulate them to build covered gallery systems from the nest to the food source. Major soldiers of *S. lamanianus* act in protecting the core portion of the colony, while minor soldiers are responsible for protecting the nest against predators and initiating a food search (Traniello and Leuthold 2000).

McMahan (1974) found non-aggressive behavior in large soldiers of *Nasutitermes exitiosus* (Isoptera, Termitidae) when compared to small soldiers, showing a marked polyethism of this caste in Isoptera. Evidence of termite polyethism was also observed in *Macrotermes bellicosus* and *Macrotermes subhyalinus* (Isoptera: Termitidae), in which minor soldiers of both species reacted to members of foreign colonies with different levels of aggression (Jmhasly and Leuthold 1999).

Soldier-initiated foraging is found in species of Rhinotermitinae as well as in some Nasutitermitinae and Termitinae (Omo Malaka and Leuthold 1986; Schedel and Kaib 1987; Wolfrum and Kaib 1988; Miura and Matsumoto 1995; Reinhard et al. 1997). In these termites, besides the role of soldiers in alarm and defense, they participate in the foraging activities and are the first to explore the unknown territories. In this context, it is possible that the presence of *H. tenuis* soldiers in the early phase of foraging activities may represent an adaptation to reduce predation during the exploratory period, which includes the discovery of food and the construction of shelter galleries, as has been reported for some *Nasutitermes* species (Traniello 1981; Cornelius and Grace 1997).

Foraging populations in these three field colonies of *H. tenuis* were estimated by mark-recapture methods as ranging from 135,000 to 640,000 individuals (Costa-Leonardo 2002). According to the methodology used in the present study, no differences were observed between the percentage of major and minor soldiers in the foraging territories of this termite species. Both workers and soldiers initiated the search for food when the percentage of soldiers in the test chambers was the same as found in the foraging territories of the field colonies. However, when the percentage of soldiers was increased, only minor soldiers were observed initiating the foraging activities. This suggests that the early phase of foraging in this species may be regulated by the number of soldiers present in the foraging territories.

The results obtained in this study suggest that there is caste polyethism in the foraging activities of *H. tenuis* under laboratory conditions. However, it remains unknown whether this also occur when this species forages underground. Further studies are also necessary to investigate caste-specific differences of the trail pheromone of this species.
